# Supplementary material for: A non-canonical role for the EDC4 decapping factor in regulating MARF1-mediated mRNA decay
Source: eLife. 2020 Jun 8;9:e54995. doi: 10.7554/eLife.54995 (PMC7279887; doi:10.7554/eLife.54995)
Supplement: Figure 3—source data 1. — (A) Sequence alignment of conserved amino acids within the MARF1 LOTUS domains 1 through 6 of human (Hs), Drosophila melanogaster (Dm), Xenopus tropicalis (Xt) and Danio rerio (Dr). LOTUS domain amino acids are in bold font. (B) Sequence alignment of human LOTUS domains 3 and 5. Identical amino acids are denoted with an asterisk and conservative amino acids substitutions are denoted with a colon. [file elife-54995-fig3-data1.pdf]

A

```
>LOTUS1
Dm    ----GSSYAKVTVPDLYFAKQVVSNLHRKKIGHKRMLVSYTRDSSLTEVNTLRCQVAGLL    316
Dr    PHTDYQLKARVHMSSLQQAISAVSLLHRYKIGSKRIQVSLVTGASNKSLTCLSSEIISIL    663
Hs    PHTDYQLKAVVQMENLQDAIGAVNSLHRYKIGSKKILVSLATGAASKSLSLLSAETMSVL    887
Xt    PHTDYQLKATVQMENLQEAICAVNSLHRYKIGSKRIQVSLATGATNKSLSLLSFGTVSIL    875
              * * :  *  *   *  *** *** *:: **    ::   :  *      :*

Dm    KDVPFNTLPMYKFRELFQSRFKTSISVLDLYKMQDICTINSDNNEEKFISLNPELVN---    373
Dr    QDAPANCLPLYKFTETYERKFGHKLAVGDLYRLPELVCVREQGGGRLVCLLSNTQIRQSP    723
Hs    QDAPACCLPLFKFTDIYEKKFGHKLNVSDLYKLTDTVAIREQGNGRLVCLLPSSQARQSP    947
Xt    QDAPACCLPLFKFTEIYEKKFGHKLIVSDLYRLTDTVTIRDQGNGRLVCLLPSVQARQSP    935
      :* *   **::** : :: :*   : * ***:: :   :  :        *

>LOTUS2
Dm    ISEIQKLIYPLLKVHTGDIPVATLLHCVKEELNVSIMAN----ENGVNLEHLICCVQGIQ    473
Dr    LKVLAAHVHSLLQSHEGMLPLLSFPDCYAAEFSALSVAEEGQLEGSVPLEHLITCIPGVT    840
Hs    LKTFAPQVHSLLQTHEGTVPLLSFPDCYIAEFGDLEVVQENQ--GGVPLEHFITCVPGVN    1061
Xt    LKTFAPQVHSLLQTHEGTVPLLSFPDCYAAEFSALKEVQEGQ--GGVPLEHLITCIPGVN    1049
      :  :   :: **: * * :*: ::  *   *:       :       * ***:* *: *:

Dm    VRANNFGIKILGWLEINKEMQSGTFNASSNTCSLTASDRTNCGSYFKNSV-ADPLFQISR    532
Dr    IVTAQNGFKVIKWIHNKPPPPNADL--------WLQ--------RSKSPVGNPQLIQFSR    884
Hs    IATAQNGIKVVKWIHNKPPPPNTDP--------WLL--------RSKSPVGNPQLIQFSR    1105
Xt    IAFAQNGIKVVKWIHNKPPPPNSDP--------WLL--------RSKSPVGNPQLIQFSR    1093
      :   : *:*:: *:  :                             *  *    *:*:**

>LOTUS3
Dm    VRANNFGIKILGWLEINKEMQSGTFNASSNTCSLTASDRTNCGSYFKNSV-ADPLFQISR    532
Dr    IVTAQNGFKVIKWIHNKPPPPNADL--------WLQ--------RSKSPVGNPQLIQFSR    884
Hs    IATAQNGIKVVKWIHNKPPPPNTDP--------WLL--------RSKSPVGNPQLIQFSR    1105
Xt    IAFAQNGIKVVKWIHNKPPPPNSDP--------WLL--------RSKSPVGNPQLIQFSR    1093
      :   : *:*:: *:  :                             *  *    *:*:**

Dm    EVIELLKMSPKSTMKFNRFIPAYHNHFGKQCRVADYGYTKLIELFEALSNVVQIMGDGEN    592
Dr    EMVDLMRSQPSCLMPVSRFIPAYHHHFAKQCRVSDYGYTKLLELLEAVPHVLQILGLGSK    944
Hs    EVIDLLKSQPSCVIPISHFIPSYHHHFAKQCRVSDYGYSKLIELLEAVPHVLQILGMGSK    1165
Xt    EVIDLLKNQPSCIMPVTKFIPTYHHHFAKQCRVSDYGYSKLLELLEAVPHVLQILGMGSK    1153
      *:::*::  *   :   :***:**:** *****:****:**:**:**: :*:**:* * :

Dm    RQITLSHRIQIRRFTSDLLRVLRANGNNSVLLSQLPLVFTQTQNKTFDITDYGVCDLIDI    652
Dr    RLLTLTHRAQVKRFTQDLLKLLKMQASKQVSLQGFGSAYHWCFSRDWQVVDYGMCDLMDL    1004
Hs    RLLTLTHRAQVKRFTQDLLKLLKSQASKQVIVREFSQAYHWCFSKDWDVTEYGVCELIDI    1225
Xt    RLLTLTHRAQVKRFTQDLLKLLKSQASKQVIVREFSQAYHWCFSRDWNVTEYGVCDLVDI    1213
      * :**:** *::*** ***::*: :  : * :  :   :     : ::: :**:*:*:*:

>LOTUS4
Dm    RQITLSHRIQIRRFTSDLLRVLRANGNNSVLLSQLPLVFTQTQNKTFDITDYGVCDLIDI    652
Dr    RLLTLTHRAQVKRFTQDLLKLLKMQASKQVSLQGFGSAYHWCFSRDWQVVDYGMCDLMDL    1004
Hs    RLLTLTHRAQVKRFTQDLLKLLKSQASKQVIVREFSQAYHWCFSKDWDVTEYGVCELIDI    1225
Xt    RLLTLTHRAQVKRFTQDLLKLLKSQASKQVIVREFSQAYHWCFSRDWNVTEYGVCDLVDI    1213
      * :**:** *::*** ***::*: :  : * :  :   :     : ::: :**:*:*:*:

Dm    LDGLVSSNIVTLGAAQNGKDILISMPKRKQTNSELEKTCVFAGEMVELFQNALQYTILFQ    712
Dr    LSEIPDSTI---SVCQQDADVIISVPKRERSAEEAERTRQFGREVVDLLRHQPHFRMAFS    1061
Hs    VSEIPDTTI---CLSQQDNEMVICIPKRERTQDEIERTKQFSKDVVDLLRHQPHFRMPFN    1282
Xt    VSEIPDTTI---CVSQQDGESVISIPKRERTPEEVERTKQFSKEVVDLLRHQPHFRMPFN    1270
      :  :  : *      *:  : :* :***:::  * *:*  *  ::*:*:::  :: : *
```

```
>LOTUS5
Dm      LDGLVSSNIVTLGAAQNGKDILISMPKRKQTNSELEKTCVFAGEMVELFQNALQYTILFQ        712
Dr      LSEIPDSTI---SVCQQDADVIISVPKRERSAEEAERTRQFGREVVDLLRHQPHFRMAFS        1061
Hs      VSEIPDTTI---CLSQQDNEMVICIPKRERTQDEIERTKQFSKDVVDLLRHQPHFRMPFN        1282
Xt      VSEIPDTTI---CVSQQDGESVISIPKRERTPEEVERTKQFSKEVVDLLRHQPHFRMPFN        1270
        :  :  : *      *:  : :* :***:::  * *:*  *  ::*:*:::  :: : *

Dm      KFVRSYHYHFAYQCRLSDYGFLKLADLLDAINGLVEMKLTSDEDKKIVLSPQVARRVFAE        772
Dr      RFIPTYHHHFGRQCKLSYYGFSKLMELFEAIPDILQVLEC-GEERLLVLTEVERVKALAA        1120
Hs      KFIPSYHHHFGRQCKLAYYGFTKLLELFEAIPDTLQVLEC-GEEKILTLTEVERFKALAA        1341
Xt      KFIPSYHHHFGRQCKLTYYGFTKLLDLFEAIPDVLQVLEC-GEEKILALTEMERIKALAS        1329
        :*: :**:**  **:*: *** ** :*::**   :::     *:: : *:     : :*


>LOTUS6
Dm      KFVRSYHYHFAYQCRLSDYGFLKLADLLDAINGLVEMKLTSDEDKKIVLSPQVARRVFAE        772
Dr      RFIPTYHHHFGRQCKLSYYGFSKLMELFEAIPDILQVLEC-GEERLLVLTEVERVKALAA        1120
Hs      KFIPSYHHHFGRQCKLAYYGFTKLLELFEAIPDTLQVLEC-GEEKILTLTEVERFKALAA        1341
Xt      KFIPSYHHHFGRQCKLTYYGFTKLLDLFEAIPDVLQVLEC-GEEKILALTEMERIKALAS        1329
        :*: :**:**  **:*: *** ** :*::**   :::     *:: : *:     : :*

Dm      QCENLIRNATGNSSHCMKLEQVLVLHKKKYGYQIQPKTLGVMDMATAVELLPY-VELKKK        831
Dr      QLVKLLRA---QRDSGLPVCRLLGEYSKTFGYCLRLQDYEASTLPALLHRLCHVVKVVDT        1177
Hs      QFVKLLRS---QKDNCLMMTDLLTEYAKTFGYTFRLQDYDVSSISALTQKLCHVVKVADI        1398
Xt      QLVKLLRS---QKDSSINMPDLLTEYSKTFGYSLRLHDYDVSSVPALMQKLCHVVKIMDT        1386
        *  :*:*    :    : :  :*  : * :** :: :      : :    * : *::

Dm      --EQAIWLICHNNDHEFRFLCYRVCKYVMERDPSASVWTNVRGEVKLTKSQQFVKPIEKS        889
Dr      PEGKEVQLI---NRKSLRALTSQLLALMMS-------LPEERSDVCVEE-----------        1216
Hs      ESGRQIQLI---NRKSLRSLTAQLLVLLMS-------WEG-TTHLSVEE-----------        1436
Xt      DLGKQIQLI---NRKSLRSLTAQLLILLMS-------WDE-SSSLTVEQ-----------        1424
           : : **   * : :* *  ::   :*               : : :
```

B

```
Hs LOTUS3  LIQFSREVIDLLKSQPSCVIPISHFIPSYHHHFAKQCRVSDYGYSKLIELLEAVPHVLQILGMGSKRLLTLT
HS LOTUS5  TKQFSKDVVDLLRHQPHFRMPFNKFIPSYHHHFGRQCKLAYYGFTKLLELFEAIPDTLQVLECGEEKILTLV
             ***::*:***: ***  :*: :********* :**::: **::**:**:**:*  **:*  * :::***
```

Figure 3-source data 1
